# Contrasting nutrient utilization strategies of dominant tree species in representative artificial and natural forests on the coastal sandy land of northern Hainan Island

**DOI:** 10.3389/fpls.2025.1640197

**Published:** 2025-11-20

**Authors:** Junting Jia, Yiqing Chen, Zongzhu Chen, Shaofeng Su, Zhipan Lin, Xiangling Lei, Haihui Chen, Shouqian Nong

**Affiliations:** 1 Hainan Academy of Forestry (Hainan Academy of Mangrove), Haikou, China; 2 Hainan Wenchang Forest Ecosystem Observation and Research Station, Wenchang, China; 3 The Innovation Platform for Academicians of Hainan Province, Haikou, China; 4 Key Laboratory of Tropical Forestry Resources Monitoring and Application of Hainan Province, Haikou, China

**Keywords:** tropical coastal, sandy land, forest, plant leaves, nutrient elements

## Abstract

The nutrient content of plant leaves serves as a critical indicator of nutrient uptake efficiency, thereby reflecting plant nutrient utilization strategies. Previous studies have primarily focused on nutrient cycling within individual forest ecosystems or the effects of management practices in artificial forests, with limited comparative analysis between artificial and natural forests. In this study, the dominant tree species in typical artificial forest and natural forest in the coastal sandy land in the northern part of Hainan Island were taken as the research objects, compared the elemental contents of dominant tree species in different forest types and analyzed the relationships among these elements. The results showed that: (1) the elements of nitrogen, phosphorus, and potassium in the leaves of artificial forest plants in northern Hainan’s coastal sandy land are 10.16%, 24.72% and 25.00% higher than those in the natural forest, respectively; the calcium content in leaves of natural forest plants is significantly higher than those in the artificial forest by 268.94%, and the elements of magnesium in the leaves of plants in the natural forest are higher than those in the artificial forest by 114.76%. (2) The correlation between the concentration of chemical elements of plant leaves in the artificial forests was stronger than that between the concentration of chemical elements of natural forests. N and K show a strong positive correlation. P is significantly positively correlated with K and Mg. Ca is highly correlated with K and Mg. N/P is negatively correlated with P and Mg. (3) Plant samples from the artificial forest had higher scores on variables related to “N” and “P”, while plant samples from the natural forest had higher scores on variables related to “Ca” and “Mg”. Overall, our results suggest that the dominant tree species in artificial forests may adopt a rapid-growth strategy, while the dominant tree species in natural forests may adopt a slow-growth strategy, and the results of this study will provide scientific and technological support for the effectiveness of vegetation restoration and evaluation of tropical coastal sand lands.

## 1 Introduction

Coastal sandy ecosystems, formed by the weathering of coastal sediments into sandy plains, extend inland from coastal beaches. These ecosystems are structurally simple and characterized by loose sandy soils, poor water-holding capacity, extremely poor nutrient availability, and high levels of salinization (Shan and Yu, 2008; Su, 2023). Due to their physical properties, they exhibit high sensitivity and responsiveness to external environmental disturbances. Despite occupying only about 3% of Hainan Island’s total land area, these ecosystems play a crucial role in coastal protection and serve as vital components of the island’s carbon cycle owing to their unique geographic positioning and carbon sequestration potential (Herrmann et al., 2015). Over the last few decades, China has undertaken significant efforts to rehabilitate and conserve coastal sandy vegetation degraded by desertification.

Engineering measures are an important means of preventing sandstorms on sandy coasts. In areas with poor vegetation coverage, strong sandstorm activity, and serious impacts on coastal production and living activities, the effect of adopting engineering sand prevention measures is more significant (Zhang and Yang, 1992). Common engineering measures include setting up sand barriers, windbreak nets, sand fixation nets, and wave-resistant sand barriers, which utilize physical facilities to block and stabilize sandstorms. A team has comprehensively adopted engineering measures such as wave prevention and sand blocking embankments, buffer zones, and sand blocking zones on tropical sandy beaches, and for the first time, has constructed a comprehensive protection system that integrates wave prevention, sand blocking, sand fixation, and sand transport, effectively ensuring the safety and sustainable functionality of national defense facilities (Qu et al., 2019).

Biological measures, with their unique ecological wisdom and sustainability, are increasingly becoming an important means of protecting coastlines and maintaining ecological balance. Introducing and optimizing biological measures, such as planting native plant communities that are tolerant to salt alkali and sandstorms, and constructing ecological protective forest belts, can not only effectively slow down wind speed and fix quicksand, but also promote soil improvement and biodiversity restoration, building a green and resilient life barrier for the coastline. The study on the protective effect of protective forests along the coast of Nansha against typhoons shows that protective forests can effectively weaken the wind speed of typhoons and reduce wind damage (Pan et al., 2014). A study in Nansha District, Guangzhou City, shows that by using a multi-level configuration of trees, shrubs, and grasses, with local tree species as the main species and some excellent introduced tree species, and adopting suitable forest management measures, an artificial forest community with certain protective functions can be constructed (Pei et al., 2013).

These large-scale ecological restoration initiatives are primarily aimed at enhancing the functionality and resilience of these fragile systems through measures such as sand stabilization, prevention of soil salinization, and mitigation of typhoon-induced damage.

The content of chemical elements in plant leaves play an important role in the plant growth, development, and morphological formation. Among the most essential elements are nitrogen (N), phosphorus (P), potassium (K), calcium (Ca), and magnesium (Mg). N and P are primary macronutrients and are widely recognized as key limiting factors in terrestrial ecosystems (Zhang et al., 2010). N, as a structural component of proteins and nucleic acids, is also involved in chlorophyll synthesis within chloroplasts, thereby directly influencing photosynthetic capacity and plant productivity (Field and Mooney, 1986; Peng and Chen, 2008; Zhou and Cao, 2021). P, an important component of nucleic acids and enzymes, is crucial for cell division, proliferation, and various physiological processes including genetic expression and mutation, which collectively enhance plant resilience (Dawson and Curran, 1998; Lambers et al., 2008; Chen and Ouyang, 2020). K plays an important role in stomatal regulation, photosynthesis, and water uptake in plants and is an essential element for maintaining plant growth, acclimatizing to cold, and improving water use efficiency, which can help the plant resist abiotic stress conditions and also promotes N uptake (Wang et al., 2013; Tripler et al., 2006; Sardans et al., 2012; Wang et al., 2024). Ca functions as a major regulator of plant development and is a structural constituent of cell walls. It also maintains membrane stability and intracellular ionic homeostasis, while acting as a key signaling molecule in response to environmental stimuli (Hepler, 2005; Al-Whaibi et al., 2010; Gilliham et al., 2011; Dodd et al., 2010; Dayod et al., 2010). Mg, the central atom in the chlorophyll molecule, also plays a bridging role in ribosome assembly, supporting N uptake and ensuring the structural integrity required for protein synthesis. Furthermore, Mg serves as a cofactor for numerous enzymes, making it indispensable for protein biosynthesis, carbohydrate metabolism, and nucleic acid processing, all of which profoundly impact plant physiological performance and growth (He and Huang, 1987; Zhou and Shen, 2013; Trnkner et al., 2018).

Diverse tree species and various forest types exhibit marked disparities in their nutritional needs and absorption capabilities. These differences not only affect plant growth and reproduction but are also fundamental to maintaining forest ecosystem health and stability. The chemical element content of plants depends on the biological characteristics of the plant species and the environment in which the plants grow. Related studies have compared foliar elemental concentrations across vegetation types, reporting relatively high macronutrient levels concentration of chemical elements in secondary young forests, scrub, mangrove forests, rubber forests, and artificial forests. However, inter-vegetation differences in major nutrient contents were generally less than twofold, with the greatest differences still below threefold (Dongsheng and Lin, 2003). By comparing the nutrient content of plant leaves from karst forests, mangrove forests on saline soils, broadleaf evergreen forests on acidic soils, and compared the stoichiometric ratio of plant elements in each habitat with the element data of plants and terrestrial higher plants in China, some studies have found that there are significant differences in the way that elements are taken up and utilized by the plants on the substrates of different habitats and that plants of specific habitats are enriched in different elements (Cui et al., 2020). Additionally, stoichiometric characteristics of foliar nutrients vary across tree species and plant functional groups. For example, research conducted in Tiantong national forest park demonstrated significant variation in nutrient use efficiency among different plant life forms (Wang et al., 2004). To cope with nutrient limitation, supply imbalances, and environmental fluctuations, vegetation adopts diverse N and P utilization strategies to optimize nutrient efficiency (Bo et al., 2000). A study (Han et al., 2021) found that fast-growing Longan species enhanced protein turnover by allocating more P to nucleic acids under P-deficient conditions, even though their total foliar P concentrations were not substantially higher than those of slow-growing counterparts. In summary, nutrient utilization strategies in plants constitute a complex ecological process by multiple factors, including species identity and forest types. Studies have shown that artificial forests of *Lindera communis* grow faster in the early stages of height growth, while natural forests grow extremely slowly in the early stages of height growth. The peak of height growth occurs 27 years later than artificial forests, indicating that the growth rate of artificial forests is earlier and faster than natural forests (Liu, 2006). Related studies have investigated different forest types on the Qinghai Tibet Plateau, and the results show that natural forests have the highest carbon content, while artificial forests have the highest nitrogen and phosphorus content, indicating that artificial forests have the fastest growth rate, highest photosynthetic efficiency, and lowest nutrient utilization efficiency (Tang et al., 2025).

However, few studies have systematically compared nutrient strategies of dominant species between artificial and natural forests on coastal sandy land. A deeper understanding of these adaptive mechanisms will improve our comprehension of plant functional ecology and provide a scientific foundation for the sustainable management and ecological restoration of forest resources in fragile coastal environments. In this study, representative artificial and natural forests located in the Wenchang region of northern Hainan Island were selected as research sites. Within each forest type, three 20 m × 20 m plots were established to conduct vegetation community surveys. Based on these surveys, three individuals of the dominant tree species in each plot were selected for analysis of foliar nutrient concentrations, focusing on five key elements: N, P, K, Ca, and Mg. The objective was to compare nutrient element dynamics across forest types and to explore species-specific nutrient utilization strategies.

Based on the ecological differences between artificial forests and natural forests in the coastal sandy areas of northern Hainan, as well as the characteristics of soil nutrient scarcity and weak water and fertilizer retention capacity in the region, the following hypotheses are proposed for the objective of this study: 1. Due to the fact that artificial forests typically receive concentrated nutrient inputs (such as fertilization) and are mainly composed of fast-growing tree species, these conditions promote the enrichment of nutrients (N, P, K) closely related to photosynthesis and growth in the leaves. On the contrary, natural forests undergo long-term natural succession on barren sandy soil, and their dominant tree species may enhance tissue stability and stress resistance by accumulating structural elements such as Ca and Mg. Therefore, we predict that: The leaf N, P, and K concentrations of dominant tree species in artificial forests will be significantly higher than those in natural forests. The leaf Ca and Mg concentrations of dominant tree species in natural forests will be significantly higher than those in artificial forests. 2. The homogenized soil environment of artificial forests (achieved through centralized management) and lower species diversity lead to species adopting similar strategies in element absorption and utilization, thereby strengthening the synergistic relationship between leaf elements. In natural forests with high species diversity and environmental heterogeneity, complex interactions between different ecological niches and species (such as competition and mutual benefit) can lead to diversified element combination strategies, thereby weakening the overall correlation between elements at the community level. Therefore, we predict that at the community level, the correlation strength between elements in artificial forest leaves will be significantly higher than that in natural forests. 3. In environments with abundant resources (such as artificial forests), plants tend to adopt a “fast-growing” strategy, prioritizing investment in high photosynthetic rates and rapid growth; In resource poor environments (such as natural forests), plants tend to adopt a “slow growth” strategy, investing more resources in organizational protection and durability. The differences in this strategy will be reflected in a series of leaf functional traits. Therefore, we predict that in principal component analysis, artificial forests have higher scores in N and P elements, while natural forests have higher scores in Ca and Mg elements.

This research aims to provide a scientific foundation for the ecological conservation and sustainable management of secondary forests on tropical coastal sandy land. Furthermore, it offers valuable references for future investigations into the growth characteristics, nutrient storage patterns, and biogeochemical cycling processes of vegetation within sandy coastal ecosystems.

## 2 Materials and methods

### 2.1 Study area

**Figure 1:**
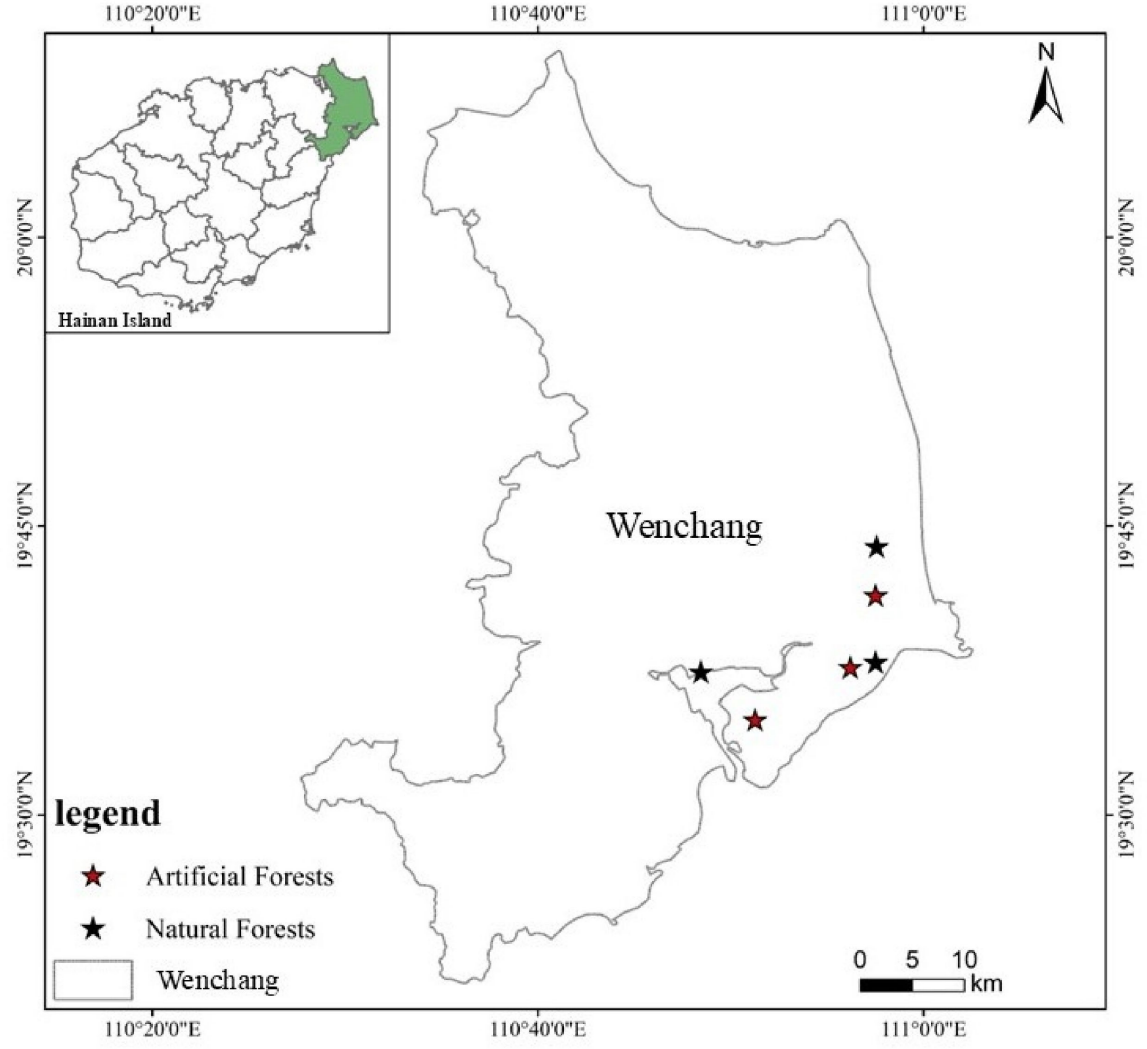
Geographic location of study site.

The study area is located in Wenchang city, Hainan province (110^°^48’-110^°^58’E and 19^°^34’-19°44’N) (**Figure 1**). The region is characterized as a coastal sandy plain and experiences a tropical oceanic monsoon climate, with mild temperatures and a distinct wet–dry seasonal pattern. The average annual temperature is 23.9°C, with an average annual sunshine duration of 1953.8 hours, an average annual precipitation of 1886.2 mm, and a mean relative humidity of 87%. The dominant vegetation type in the area is secondary forest, and the soil type is classified as coastal sandy soil. The slope of the sample plot is all less than 5°. The elevations of natural forest plots are 24.9 meters, 5.6 meters, and 0.2 meters, respectively, while the elevations of artificial forest plots are 23.8 meters, 15.2 meters, and 26.1 meters, respectively. Although the sample plots exhibit variation in elevation, all are situated within a continuous coastal sandy plain characterized by minimal topographic relief (slope*<*5°) and high homogeneity in both soil type and vegetation structure. To quantitatively assess the potential confounding effect of elevation on nutrient dynamics, we performed a correlation analysis between plot elevation and the availability of key soil nutrients. The results demonstrated no significant correlation between elevation and soil total nitrogen, total phosphorus, or total potassium within either forest type (p*>*0.05). This analysis confirms that the observed elevation gradient is not a significant driver of soil nutrient availability in this homogeneous coastal ecosystem.

### 2.2 Tree species

To investigate the structural characteristics of forest communities in the study area and identify dominant tree species, sample plots representing artificial and natural forest types were established in Wenchang city. Based on field investigations within these plots, the abundance of each species was calculated, and combined with their ecological importance, ten dominant tree species were identified for subsequent analysis (**Table 1**). All studied tree species are evergreen species with no obvious concentrated leaf shedding period.

**Table 1:** The 10 main tree species selected within the two forest types.

Forest types	Species	Code	Family	Genera
Artificial Forest	*Cocos nucifera* L.	Coc nuc	Arecaceae	*Cocos*
	*Litsea glutinosa* (Lour.) C. B. Rob.	Lit glu	Lauraceae	*Litsea*
	*Castanopsis wenchangensis* G. A. Fu & C. C. Huang	Cas wen	Fagaceae	*Castanopsis*
	*Heteropanax fragrans* (Roxb.) Seem.	Het fra	Araliaceae	*Heteropanax*
	*Litsea pseudoelongata* H. Liu	Lit pse	Lauraceae	*Litsea*
Natural Forest	*Calophyllum inophyllum* L.	Cal ino	Calophyllaceae	*Calophyllum*
	*Talipariti tiliaceum* (L.) Fryxell	Tal til	Malvaceae	*Talipariti*
	*Garcinia oblongifolia* Champ. ex Benth.	Gar obl	Clusiaceae	*Garcinia*
	*Myrsine linearis* (Lour.) Poir.	Myr lin	Primulaceae	*Myrsine*
	*Psychotria asiatica* L.	Psy asi	Rubiaceae	*Psychotria*

### 2.3 Leaf blade sample collection

In July 2022, within each forest type, three 20 × 20 m plots were randomly established to survey woody vegetation with a diameter at breast height (DBH) ≥ 1 cm. After vegetation investigation and relative dominance analysis, five individuals of each dominant tree species were selected from each plot for leaf sampling. The selection is based on the following objective criteria to ensure that the sampled individuals represent the average status of species within the plot: healthy, mature, representing the average size of the population within the plot. This standardization scheme aims to minimize sampling bias by avoiding individuals with developmental delays, excessive maturity, or abnormally strong traits. From each individual, five fully expanded, mature leaves in their stable growth phase were randomly sampled from sun-exposed branches in the mid-to-upper canopy. Sampling was conducted during the peak growing season. Leaf samples were collected from the fully expanded branches located in the mid- to upper outer canopy, placed in sealed plastic bags, and transported to the laboratory for elemental analysis. Fresh leaf samples were initially air-dried in a well-ventilated area and subsequently oven-dried at 80 ^°^C to remove residual moisture.

### 2.4 Measurement indicators and methods

The determination indexes of leaf samples, including leaf N, P, K, Ca, and Mg, were determined. Among them, the total N of plant leaves was first determined by H_2_SO_4_-H_2_O_2_ digestion and then by the semi-micro distillation method. The total P of the plant was first determined by H_2_SO_4_-H_2_O_2_ digestion and then by molybdenum and antimony anti-absorbent spectrophotometry. The total K of the plant was first determined by H_2_SO_4_-H_2_O_2_ digestion and then by the flame spectrophotometer method. The total Ca and Mg of plants were first determined by HNO_3_-HClO_4_ digestion and then by atomic absorption spectrophotometry. The digestion methods were selected based on the distinct chemical properties of the target elements and established analytical protocols in plant nutrient analysis. The H_2_SO_4_-H_2_O_2_ digestion system is highly effective for the oxidation of organic matter and the conversion of nitrogen and phosphorus into soluble forms suitable for subsequent colorimetric analysis. It also provides a suitable matrix for the precise flame photometric determination of potassium. In contrast, the HNO_3_-HClO_4_ digestion system, involving stronger oxidizing acids, is required for the complete mineralization of plant tissue and the effective release of mineral cations like calcium and magnesium into solution. This method prevents the formation of insoluble sulfate precipitates, which can occur in a sulfate-rich matrix and would negatively impact the accuracy of atomic absorption spectrophotometry for Ca and Mg. The use of these two well-established, element-specific digestion procedures is a standard practice in ecological stoichiometry and ensures optimal recovery and accurate measurement for each group of elements. (1) H_2_SO_4_–H_2_O_2_Digestion: Mix concentrated H_2_SO_4_ (98%) with H_2_O_2_ (30%) in a volume ratio of 5:1. Place approximately 0.2g of dried and finely ground leaf sample into a digestive tube. Add 5 mL of concentrated H_2_SO_4_ into each test tube. After mixing the sample with the digestion solution, heat the test tube for 30 minutes and add H_2_O_2_ dropwise until the solution is colorless and transparent. After cooling, dilute to 50mL for subsequent measurement. (2) HNO_3_–HClO_4_ Digestion: Mix concentrated HNO_3_ (68%) and HClO_4_ (70%) in a volume ratio of 4:1. Place approximately 0.5g of dried leaf sample into the digestive tube. Add enough mixed acid, mixing the sample with the digestion solution, and heat until the solution becomes clear. After cooling, dilute to 50mL for subsequent measurement (Bocock, 1964). Quality control measures were rigorously implemented throughout the analytical process to ensure data accuracy and precision. These included: (1) Use of certified reference materials to verify analytical accuracy, with recovery rates maintained between 95-105% for all elements; (2) Inclusion of duplicate samples to assess analytical precision, with relative standard deviations maintained below 5% for all elements; (3) Procedural blanks were analyzed with each batch to correct for potential contamination; (4) Instrument calibration was verified to ensure measurement stability throughout the analytical sequence.

### 2.5 Data analysis

Microsoft Excel 2019 was used for basic data collation and descriptive statistical analysis. OriginPro 2022 software was employed for data processing and graphical visualization. To statistically validate the observed variability patterns in foliar nutrient concentrations, we performed Levene’s test for homogeneity of variances using SPSS (version 25). This test assesses whether the absolute dispersion of data points differs significantly between groups, providing a robust statistical basis for comparing the variability patterns indicated by the coefficients of variation (CV). One-way analysis of variance (ANOVA) was performed to assess the significance of differences in elemental contents among different tree species and forest types. To ensure statistical robustness given the observed variability in elemental distributions, we complemented the parametric ANOVA with non-parametric Kruskal-Wallis tests. This dual analytical approach was specifically adopted to verify that our conclusions were not influenced by potential violations of parametric test assumptions. Spearman’s rank correlation test was conducted to evaluate the relationships among different chemical elements. To control the family-wise error rate (Type I error) arising from multiple comparisons, the significance level for the correlation matrices was adjusted using the conservative Bonferroni correction. The adjusted significance level was set at p ≤ 0.003 (i.e., 0.05/15 comparisons) for each forest type. Additionally, elemental ratio calculations and PCA were conducted to explore stoichiometric patterns in plant leave concentration of chemical elements.

## 3 Results

### 3.1 Comparative analysis of elemental content of leaves of plants of different forest types

**Table 2** presents the mean concentrations of five key nutrients—N, P, K, Ca, and Mg—in the foliage of dominant tree species across both artificial forest and natural forests along the coastal sandy regions of northern Hainan Island. In artificial forests, the average foliar elemental concentrations followed the order: N*>*K*>*Ca*>*Mg*>*P, while in natural forests, the order was: N*>*Ca*>*K*>*Mg*>*P. When compared with the reported concentration ranges for terrestrial vascular plants, the chemical elements in the leaves of plants from the coastal sands of northern Hainan Island were all within the normal range. Compared with the content of elements required by higher plants, the average N#content of artificial forest and natural forest was close to the content of elements required by higher plants, and the average K content of artificial forest was close to the content of elements required by higher plants.

**Table 2:** Leaf concentration of chemical elements of the major dominant tree species.

Parameters	Concentration of chemical elements (g/kg)									
	N		P		K		Ca		Mg	
	Artificial	Natural	Artificial	Natural	Artificial	Natural	Artificial	Natural	Artificial	Natural
Minimum	11.32	11.1	0.51	0.22	3.31	2.98	1.05	7.11	0.82	1.49
Maximum	30.28	22.96	1.88	1.62	20.44	13.24	7.34	24.98	4.18	11.21
Median	18.11	16.16	1.10	0.77	6.49	5.23	3.59	15.22	1.67	2.90
Average	18.00	16.34	1.11	0.89	8.60	6.88	4.25	15.68	2.10	4.51
Std Dev	4.72	3.16	0.31	0.32	4.92	3.29	1.78	4.82	1.08	2.96
CV%	26.22	19.34	27.93	35.96	57.21	47.82	41.88	30.74	51.43	65.63
Max/Min	2.67	2.07	3.69	7.36	6.18	4.44	6.99	3.51	5.10	7.52

The coefficients of variation for chemical elements in artificial forests followed the order: K*>*Mg*>*Ca*>*P*>*N, while in natural forests, the order was: Mg*>*K*>*P*>*Ca*>*N. In artificial forests, the coefficients of variation for K and Mg as well as Mg in natural forests, are greater than 50%, while others are less than 50%. The ratio of the maximum value of the content of a chemical element to the minimum value indicates the size of the difference in the content of the same chemical element in the leaves of different plants, the ratios of the artificial forest are Ca*>*K*>*Mg*>*P*>*N in ascending order, with the difference of Ca, K and Mg more than 5 times, P and N less than 5 times. The ratio of natural forest is Mg*>*P*>*K*>*Ca*>*N in descending order, in which the difference between P and Mg is more than 5 times, and the difference between K, Ca, and N is less than 5 times. Among all elements, Mg exhibited the greatest range between maximum and minimum values, whereas N showed the least variation. These findings suggest species-specific patterns of elemental uptake and relatively stable nutrient content for certain elements across different tree species.

Levene’s test for homogeneity of variances provided statistical validation of these variability patterns. The results showed that Mg was the only element to exhibit statistically significant differences in variance between forest types (F = 11.323, p = 0.010), with natural forests showing significantly greater variability. This statistical finding aligns with the descriptive CV pattern (natural forest: 65.63% vs. artificial forest: 51.43%). Ca showed marginally non-significant differences in variance (F = 4.828, p = 0.059). In contrast, nitrogen (N: F = 3.173, p = 0.081), phosphorus (P: F = 0.034, p = 0.854), and potassium (K: F = 2.865, p = 0.097) showed no statistically significant differences in variance between forest types. These statistically validated findings highlight the complex nature of nutrient variability in coastal sandy ecosystems, with magnesium emerging as the element with the most pronounced and statistically verified difference in variability between forest types.

**Figure 2:**
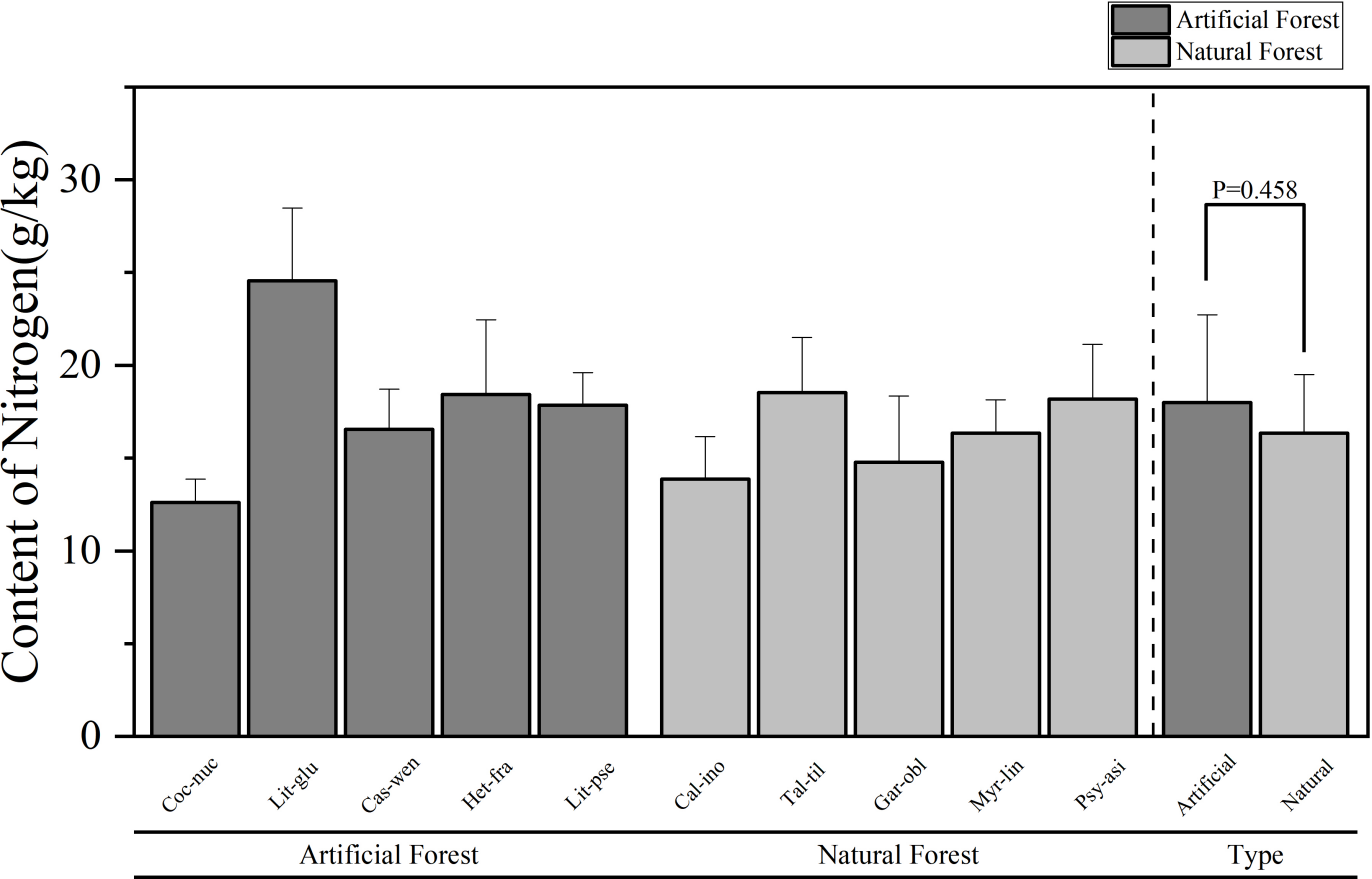
Comparison of leaf N content of plants in different forest types.

**Figure 3:**
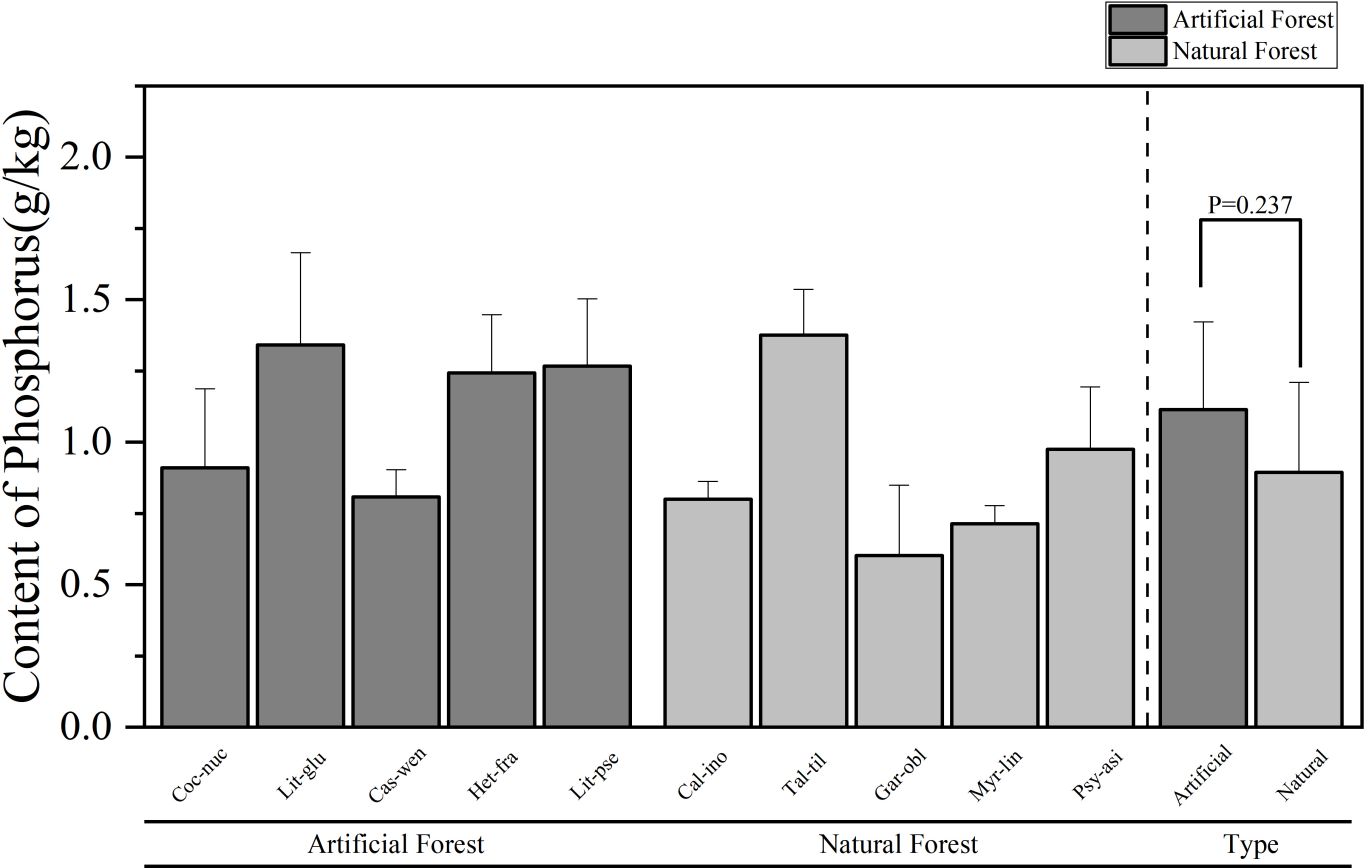
Comparison of P content of leaves of plants in different forest types.

**Figure 4:**
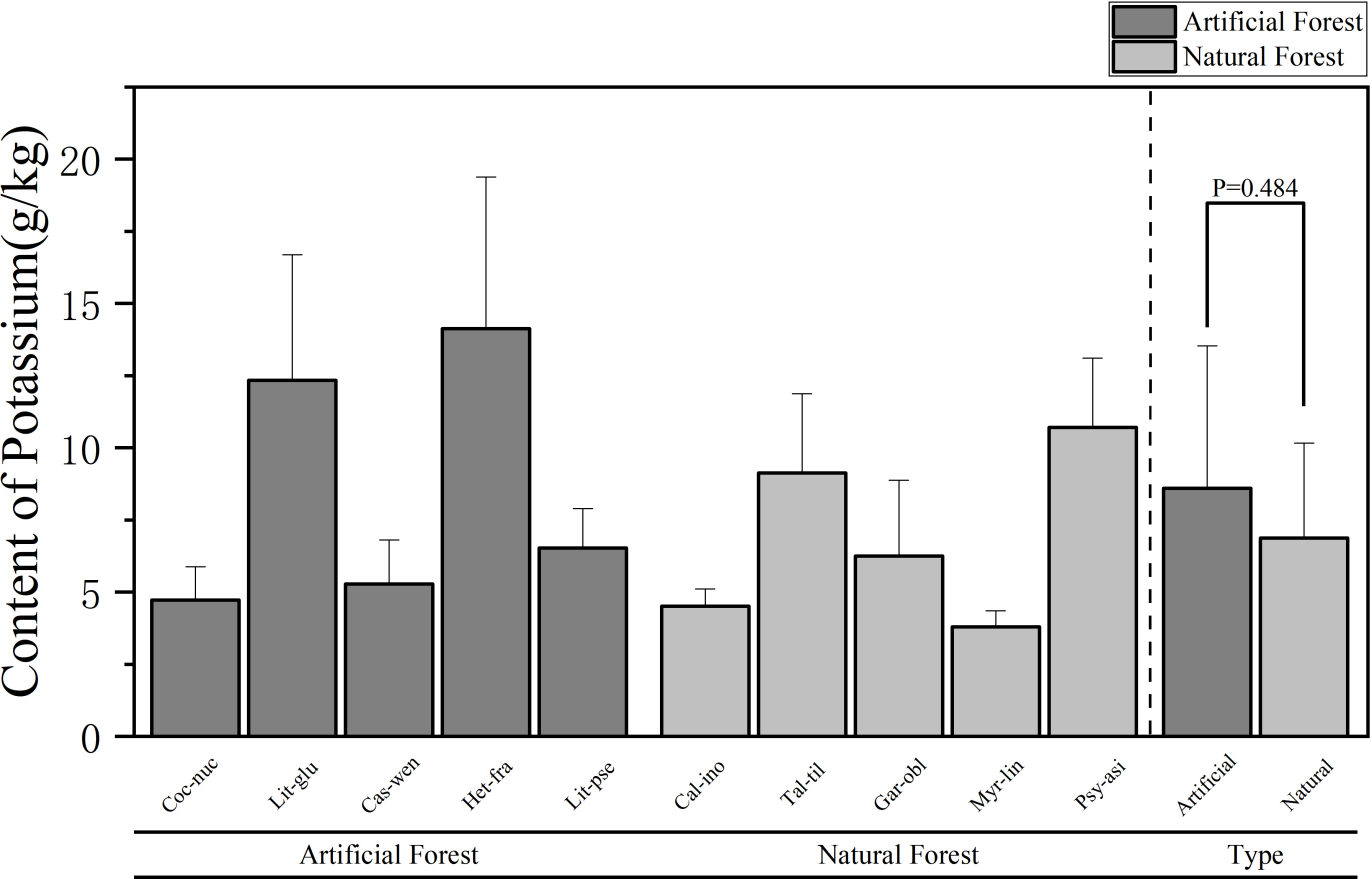
Comparison of K content of leaves in plants of different forest types.

**Figure 5:**
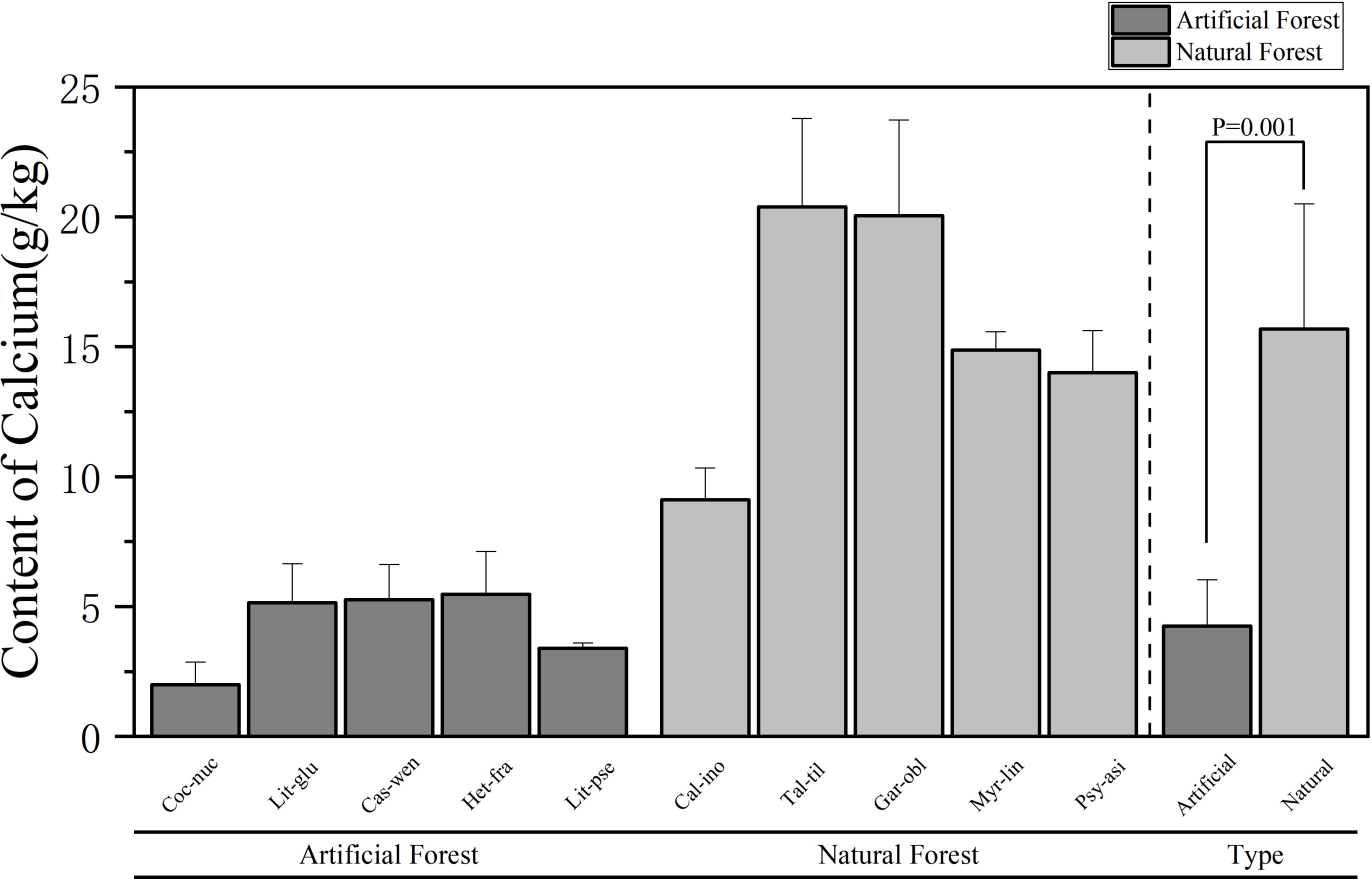
Comparison of Ca content of leaves of plants in different forest types.

A comparison of the chemical contents of leaves of plants of different forest types in coastal sandy vegetation in northern Hainan Island is presented in **Figures 2**–**5**. **Figure 2** illustrates the differences in N concentrations among the dominant species in artificial and natural forests. The average N content of artificial forest was (18.00 ± 4.72) g/kg. The average value of N content of natural forest was (16.34 ± 3.16) g/kg, and the mean N content in artificial forest was notably greater than that in natural forest. Among the 10 plants, the average N content of plant leaves in descending order is: *L. glutinosa > T. tiliaceum > H. fragrans > P. asiatica > L. pseudoelongata > C. wenchangensis > M. linearis > G. oblongifolia > C. inophyllum > C. nucifera*. The average value of N content in the leaves of *L. glutinosa* is the highest and significantly higher than that of other tree species, at (24.56 ± 3.92) g/kg. In contrast, *C. nucifera* showed the lowest foliar N content, with an average value of (12.62 ± 1.26) g/kg, significantly less than other species. Within artificial forests, *L. glutinosa* had the highest leaf N concentration, while *C. nucifera* had the lowest. In natural forest, the highest N content was observed in *T. tiliaceum*, whereas *C. inophyllum* exhibited the lowest.

A comparison of the P content of leaves of plants of different forest types is shown in **Figure 3**. The average P in leaves from artificial forests was (1.11 ± 0.31) g/kg, while that from natural forests was (0.89 ± 0.32) g/kg. Overall, artificial forest species exhibited higher average foliar P content than natural forest species. Among the 10 plants, the average content of plant leaves in descending order is: *T. tiliaceum > L. glutinosa > L. pseudoelongata > H. fragrans > P. asiatica > C. nucifera > C. wenchangensis > C. inophyllum > M. linearis > G. oblongifolia*. The average P content of *T. tiliaceum* had the highest mean P concentration (1.37 ± 0.16)g/kg, while *G. oblongifolia* exhibited the lowest (0.60 ± 0.25)g/kg. The average P content of *L. glutinosa* in the artificial forests was the highest, while the average P content of *C. wenchangensis* leaves was the lowest; in the natural forests, the average P content of *T. tiliaceum* leaves was the highest, while the average P content of leaves of *G. oblongifolia* was the lowest.

A comparison of K content of plants in different forest types is shown in **Figure 4**. The average K content in leaves of artificial forest species was (8.60 ± 4.92) g/kg, while that in natural forests was (6.88 ± 3.29) g/kg. Overall, the artificial forest exhibited a higher average foliar K content than the natural forest. Among the 10 plants, the average K content of plant leaves in descending order is: *H. fragrans > L. glutinosa > P. asiatica > T. tiliaceum > L. pseudoelongata > G. oblongifolia > C. wenchangensis > C. nucifera > C. inophyllum > M. linearis*. *H. fragrans* exhibited the highest foliar K content (14.13 ± 5.26) g/kg, significantly exceeding that of other species. In contrast, *M. linearis* had the lowest K content, at (3.79 ± 0.55) g/kg. The average K content of *H. fragrans* and *L. glutinosa* in the artificial forests was significantly higher than that of other trees. The average K content of *H. fragrans* was the highest, and that of *C. nucifera* was the lowest; in the natural forests, the average K content of *P. asiatica* and *T. tiliaceum* was significantly higher than that of other trees. *P. asiatica* exhibited the highest average K content, whereas *M. linearis* had the lowest.

A comparison of Ca content in leaves of plants of different forest types is shown in **Figure 5**. The average Ca content in artificial forests was (4.25 ± 1.78) g/kg, whereas a in natural forests it reached (15.68 ± 4.82) g/kg. Overall, natural forest species exhibited significantly higher Ca concentrations than those in artificial forests. Among the 10 plants, the average Ca content of plant leaves in descending order is: *T. tiliaceum > G. oblongifolia > M. linearis > P. asiatica > C. inophyllum > H. fragrans > C. wenchangensis > L. glutinosa > L. pseudoelongata > C. nucifera*. The average Ca content of *T. tiliaceum* leaves is the highest, (20.38 ± 3.40) g/kg. The average Ca content of *C. nucifera* leaves is the lowest, (2.00 ± 0.87) g/kg. The average Ca content of *H. fragrans* in the artificial forests was highest, and the average Ca content of *C. nucifera* was the lowest; in the natural forest, the average Ca content of *T. tiliaceum* was the highest, and the average Ca content of *C. inophyllum* was the lowest.

Comparative analysis of foliar elemental concentrations between artificial and natural forests revealed distinct patterns. To ensure statistical robustness, use parametric (ANOVA) and non-parametric (Kruskal Wallis) methods for testing, which demonstrated remarkable consistency across all elements: All other elements - N, P, K and Mg - consistently showed no significant differences between forest types in both analytical approaches (N: ANOVA p = 0.458, Kruskal-Wallis p = 0.602; P: ANOVA p = 0.237, Kruskal-Wallis p = 0.251; K: ANOVA p = 0.484, Kruskal-Wallis p = 0.347; Mg: ANOVA p = 0.118, Kruskal-Wallis p = 0.117). Ca was the only element to show statistically significant differences between forest types in both analytical frameworks (ANOVA p = 0.001, Kruskal-Wallis p = 0.009), providing strong and consistent evidence for fundamental differences in Ca accumulation strategies between artificial and natural forests.

**Figure 6:**
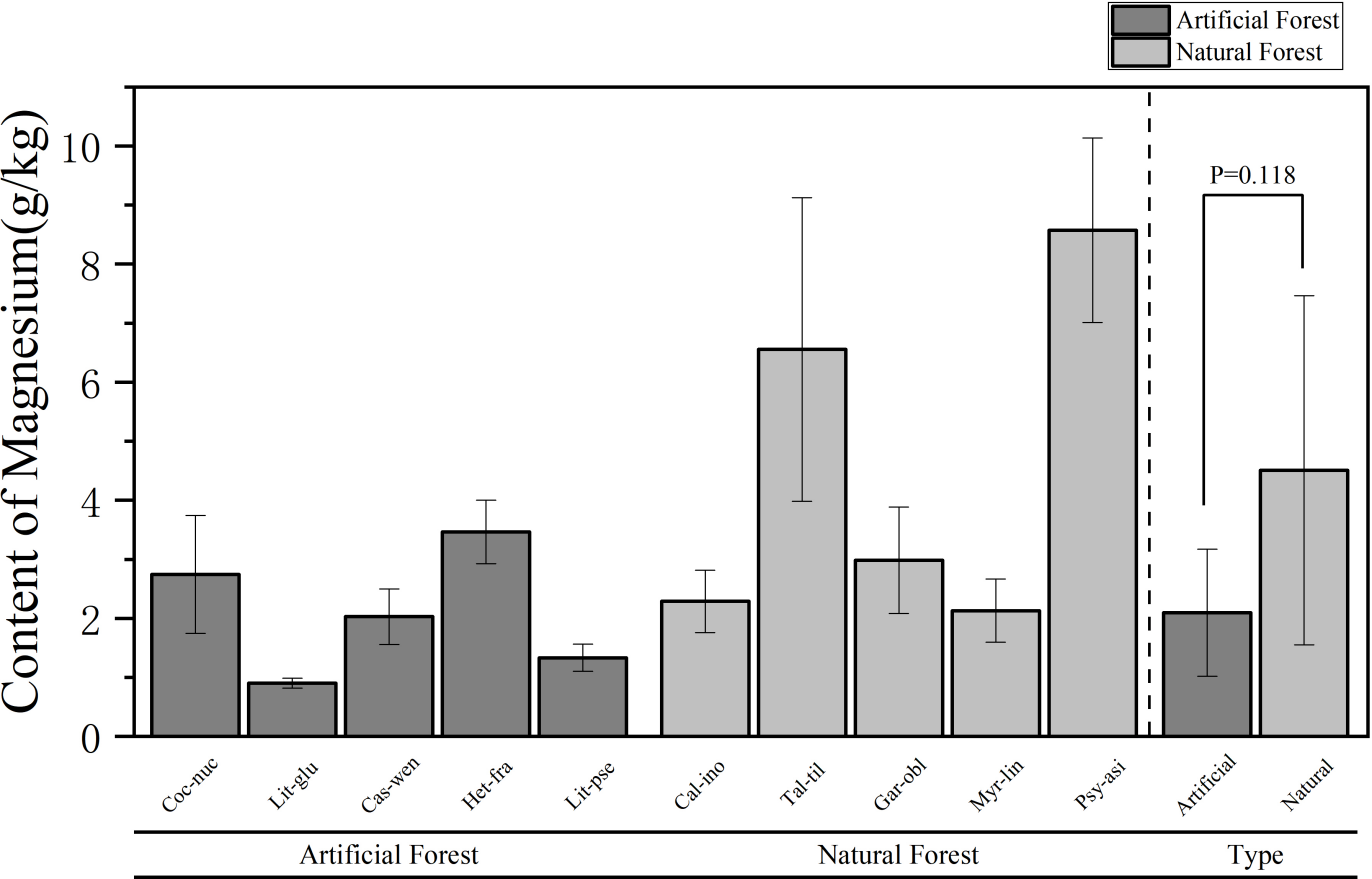
Comparison of Mg content in leaves of plants of different forest types.

A comparison of the Mg content of leaves of plants of different forest types is shown in **Figure 6**. The average Mg content in the artificial forest was (2.10 ± 1.08) g/kg, while in natural forest leaves it was (4.51 ± 2.96) g/kg. On average, natural forest species exhibited higher foliar Mg concentrations than those in artificial forests. Among the 10 plants, the average Mg content of plant leaves in descending order is: *P. asiatica > T. tiliaceum > H. fragrans > G. oblongifolia > C. nucifera > C. inophyllum > M. linearis > C. wenchangensis > L. pseudoelongata > L. glutinosa*. The average Mg content of *P. asiatica* is the highest (8.57 ± 1.56) g/kg. The Mg content of *P. asiatica* and *T. tiliaceum* is significantly higher than that of other trees. The Mg content of *L. glutinosa* was the lowest, which was (0.90 ± 0.08) g/kg. The average Mg content of *H. fragrans* in the artificial forests was the highest, and the average Mg content of *L. glutinosa* was the lowest; in the natural forests, the average Mg content of *P. asiatica* was the highest, and the average Mg content of *M. linearis* was the lowest.

### 3.2 Ratio of chemical elements in leaves of plants of different tree species

Based on the elemental concentration data of dominant species from typical artificial and natural forests in the coastal sandy land of northern Hainan Island concentration of chemical elements (**Table 3**), stoichiometric ratios of five key nutrients (N, P, K, Ca, and Mg) were calculated. The N and P ratio in plant leaves is influenced by factors such as habitat conditions, growth stage, and plant functional group. The N/P ratios are widely used as indicators of shifts in trophic structure, biodiversity, and ecosystem biogeochemical cycling. Specifically, the N/P ratio reflects the relative availability and utilization efficiency of nitrogen and phosphorus, serving as a key indicator of nutrient limitation in plants.

**Table 3:** The ratio of chemical element content in leaves of different tree species and plants.

Species	N/P	N/K	N/Mg	K/P	K/Mg	Ca/N	Ca/P	Ca/K	Ca/Mg	Mg/P
*C. nucifera*	10.58	2.83	5.29	4.08	1.85	0.16	1.90	0.47	0.87	2.34
*L. glutinosa*	13.95	2.13	27.22	6.80	13.56	0.22	3.36	0.48	5.83	0.54
*C. wenchangensis*	20.84	3.24	8.71	6.69	2.85	0.32	6.52	1.02	2.81	2.55
*H. fragrans*	12.79	1.54	5.39	11.17	4.25	0.31	3.70	0.47	1.60	2.46
*L. pseudoelongata*	12.71	2.81	13.93	4.77	5.00	0.19	2.42	0.54	2.62	0.97
*C. inophyllum*	40.73	3.11	6.47	12.25	2.05	0.68	22.53	2.04	4.11	5.19
*T. tiliaceum*	13.73	2.22	3.32	6.52	1.72	1.13	14.81	2.34	3.75	4.92
*G. oblongifolia*	33.82	2.47	5.27	15.89	2.32	1.38	44.51	3.41	7.21	5.75
*M. linearis*	23.02	4.38	7.94	5.33	1.82	0.92	21.05	4.01	7.35	2.99
*P. asiatica*	19.01	1.82	2.16	11.51	1.28	0.78	14.78	1.38	1.65	9.05
Artificial Forest	14.17	2.51	12.11	6.70	5.50	0.24	3.58	0.60	2.75	1.77
Natural Forest	26.06	2.80	5.03	10.30	1.84	0.98	23.54	2.64	4.81	5.58
Average value	20.12	2.66	8.57	8.50	3.67	0.61	13.56	1.62	3.78	3.68
Terrestrial higher plants^1^	7.5	1.5	7.5	5	5	0.33	2.5	0.5	2.5	1

^1^The data for ‘Territorial higher plants’ is referenced in ‘Plant Physiology and Development’ (Taiz and Zeiger, 2015).

To assess nutrient limitation patterns, we applied the conventional stoichiometric thresholds where N/P *<* 14 suggests N limitation, N/P *>* 16 suggests P limitation, and 14 *<* N/P *>* 16 indicates co-limitation by both elements. Among the 10 tree species in this study, N/P ratios ranged from 10.58 (*C. nucifera*) to 40.73 (*C. inophyllum*), with a mean value of 20.12. In this experiment, the N/P values of five plants, namely *C. wenchanggensis*, *C. inophyllum*, *G. oblongifolia*, *M. linearis*, and *P. asiatica*, are all greater than 16, suggesting potential phosphorus limitation; the N/P value of *L. glutinosa*, *T. tiliaceum*, *H. fragrans*, *L. pseudoelongata*, and *C. nucifera* is less than 14, suggesting potential nitrogen limitation. However, it is important to note that these threshold values were primarily established for temperate plant species and ecosystems. Tropical coastal species, particularly those adapted to sandy soil environments, may exhibit different critical N/P ratios due to species-specific physiological adaptations and nutrient use efficiencies. Therefore, while these conventional thresholds provide useful preliminary insights, and future studies incorporating nutrient addition experiments would be necessary to validate these nutrient limitation patterns for our specific study system.

Per Venterink et al.’s thresholds: K-limited growth occurs when plant K/P <3.4 and N/K >2.1; P-limited when K/P >3.4; and N-limited when N/K <2.1. In this study, all ten examined species exhibited K/P ratios greater than 3.4, indicating that none of the species were limited by K.

As shown in **Table 1**, compared with terrestrial higher plants, the nine pairs of nutrient element ratios of N/P, N/K, N/Mg, K/P, Ca/N, Ca/P, Ca/K, Ca/Mg, and Mg/P in the leaves of plants from the coastal sandy vegetation in the northern part of Hainan Island were greater for terrestrial higher plants, yet the K/Mg element ratios were smaller in comparison. The fact that the ratios of N/P, N/K, N/Mg and Ca/N were higher than those of terrestrial higher plants showed that the plant leaves were more deficient in N than Ca, while N was more abundant than P, K and Mg; the ratios of K/P, Ca/P and Mg/P were higher than those of terrestrial higher plants, indicating that the plant leaves were more deficient in P; the ratios of K/Mg were lower than those of terrestrial higher plants, and the ratios of Ca/K were higher than those of terrestrial higher plants, indicating a relative deficiency of K element in the leaves compared to Ca and Mg elements; Ca/Mg is higher than that of terrestrial plants, which means that Ca is more abundant than Mg. Overall, the relative nutrient availability in the leaves of coastal sandy vegetation in northern Hainan Island followed the order: Ca *>* N *>* Mg *>* K *>* P. This pattern reflects an environmental condition characterized by relative sufficiency of Ca, N, and Mg, and a marked deficiency of K and P.

### 3.3 Correlation analysis of concentration of chemical elements of plant leaves

**Figure 7:**
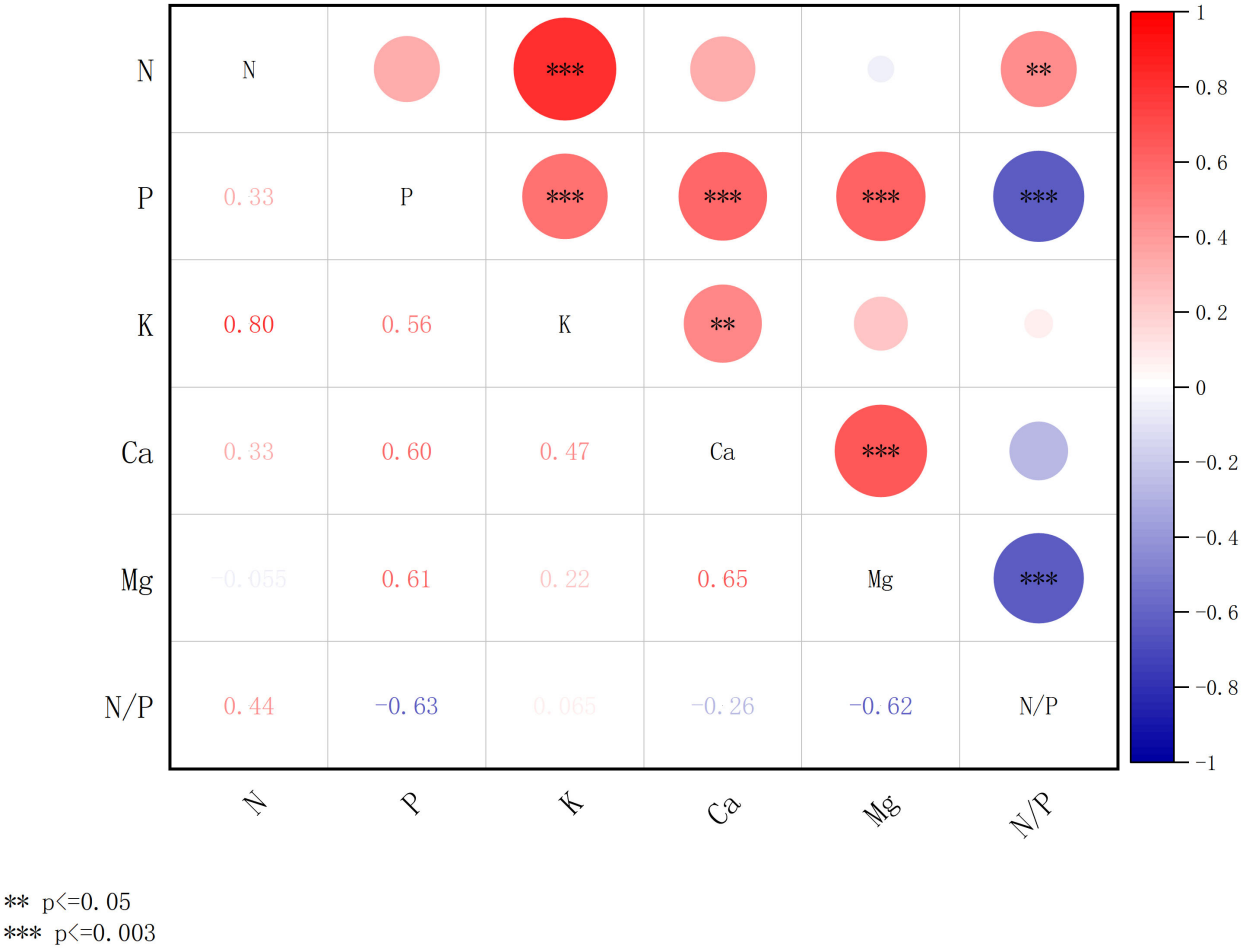
Correlation of concentration of chemical elements of plant leaves in the artificial forests. *** denotes significance after Bonferroni correction (p ≤ 0.003), and ** denotes significance at the nominal level (p ≤ 0.05) prior to correction.

**Figure 8:**
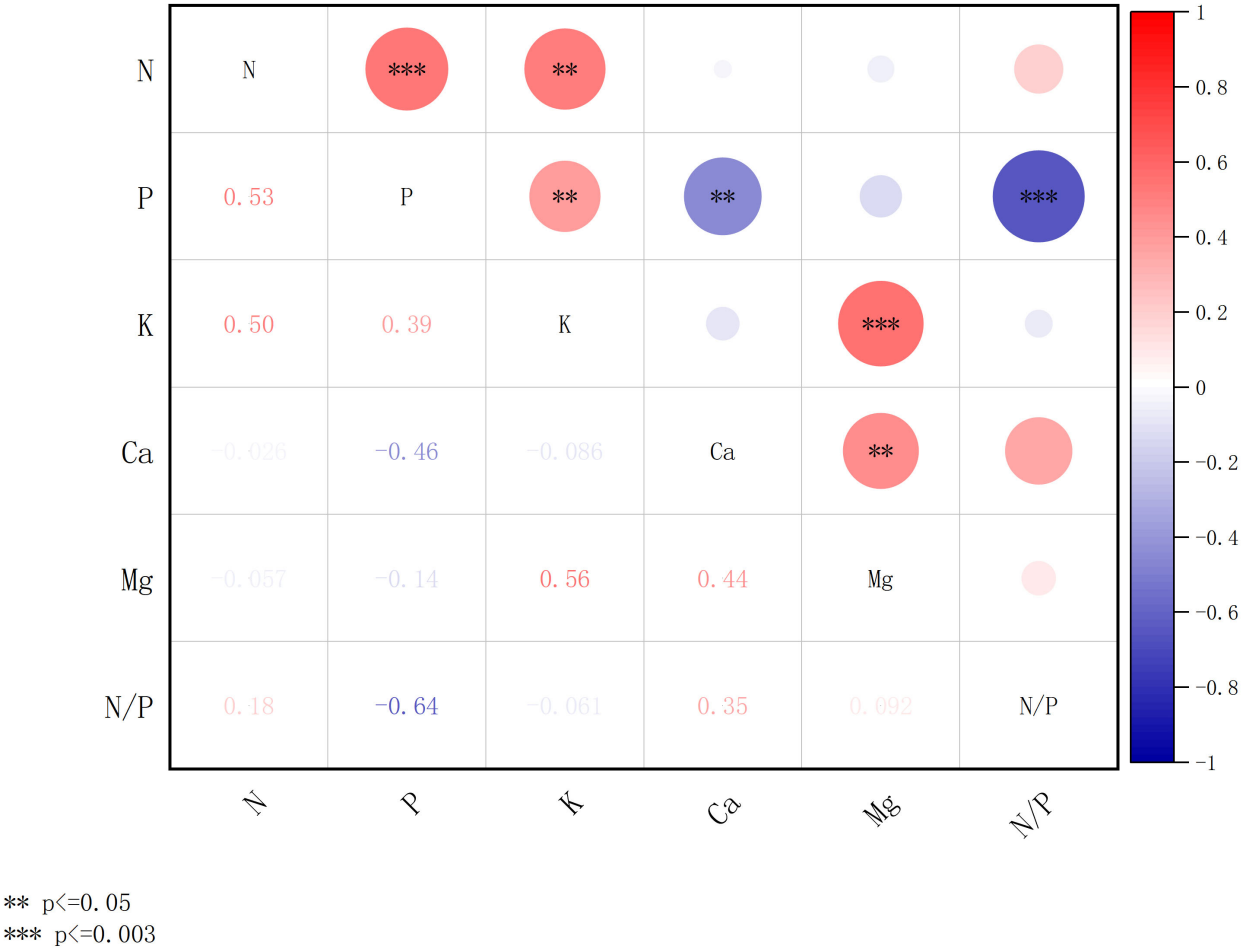
Correlation of concentration of chemical elements of plant leaves in the natural forests. *** denotes significance after Bonferroni correction (p ≤ 0.003), and ** denotes significance at the nominal level (p ≤ 0.05) prior to correction.

**Figures 7**, **8** present the results of a comparative correlation analysis of foliar nutrient concentrations among dominant tree species in both artificial forests and natural forests within the coastal sandy ecosystems of northern Hainan Island. The study examines the interrelationships between various mineral nutrients across these distinct vegetation types.

**Figure 7** illustrates the interrelationships among foliar nutrient concentrations in artificial forest vegetation. The concentrations of N, P, K, Ca, and Mg exhibited varying degrees of correlation. Notably, N and K demonstrate a strong, positive relationship (p ≤ 0.003), with a correlation coefficient hitting a solid 0.80. There is a highly significant positive correlation (p ≤ 0.003) between the content of the P element and the content of K, Ca, and Mg elements, with correlation coefficients of 0.56, 0.60, and 0.61, respectively. There was a highly significant positive correlation (p ≤ 0.003) between the content of element Ca and the content of elements Mg, with correlation coefficients of 0.65. N/P, in addition to being correlated with element P, also showed a significant negative correlation (p ≤ 0.003) with the element Mg, with correlation coefficients of -0.63 and -0.62, respectively.

**Figure 8** shows the correlation of concentration of chemical elements in leaves of natural forest plants. The concentrations of N, P, K, Ca, and Mg showed varying degrees of interrelationships. A highly significant positive correlation was observed between N and both P, with correlation coefficients of 0.53 (p ≤ 0.003). Additionally, K was positively and significantly correlated with Mg (r = 0.56, p ≤ 0.003). The N/P ratio showed a significant negative correlation with P (r = -0.64, p ≤ 0.003).

**Figure 9:**
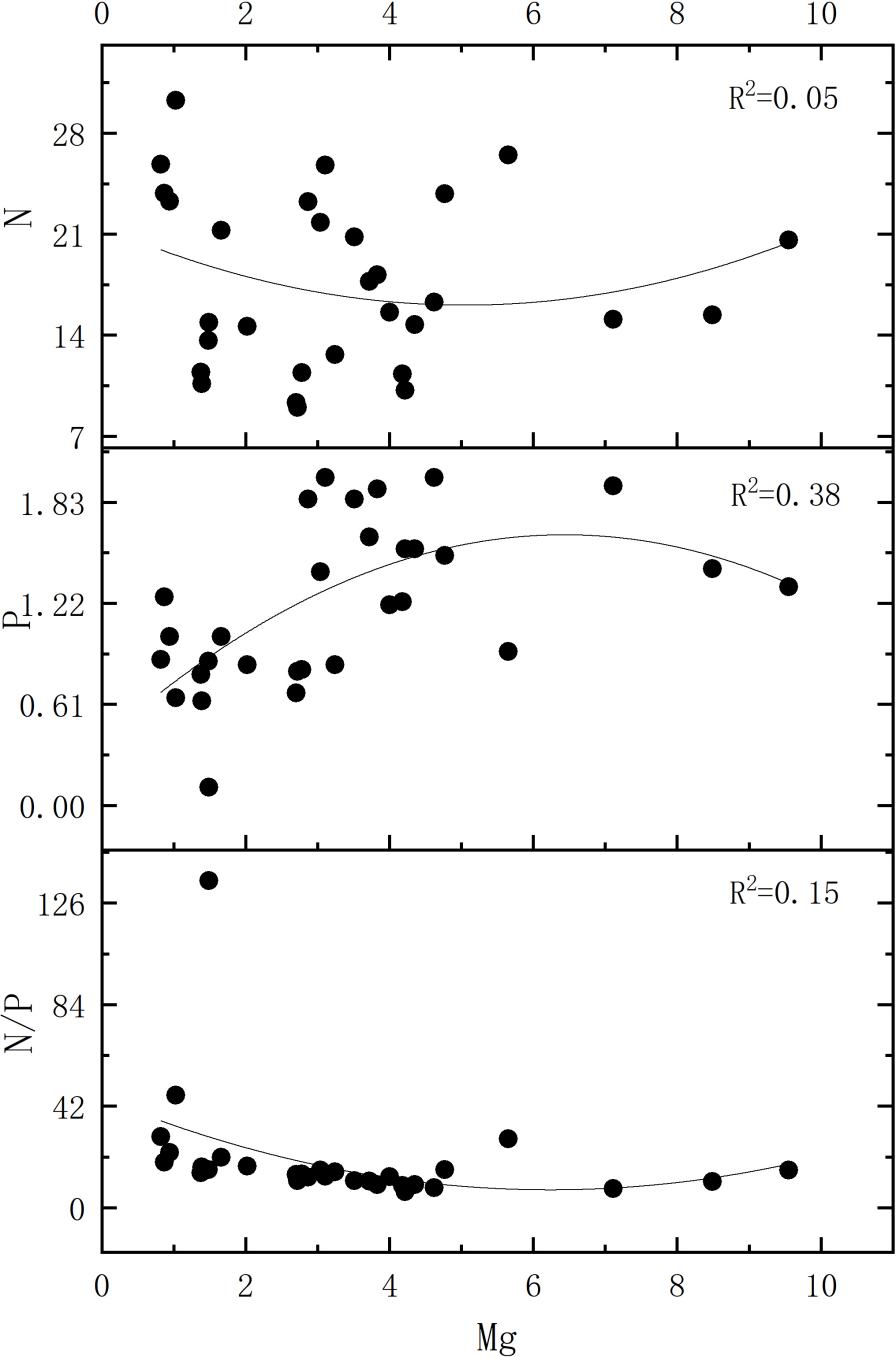
Correlation of leaf N, P, and N/P ratio with Mg content in the artificial forest plants.

**Figures 7**, **8** collectively indicate stronger correlations among foliar chemical elements in artificial forests compared to natural forest. As illustrated in **Figure 7**, a distinct relationship was observed between the N/P ratio and Mg concentration in artificial forest leaves. To further explore this pattern, **Figure 9** presents the quantitative relationship between foliar N and P contents, the N/P ratio, and Mg concentration in artificial forest species. As illustrated, the N/P ratio initially decreases with increasing Mg concentration until reaching a minimum when Mg content equals 6.24 g/kg. (This turning point was determined using the regression equation derived in **Figure 9**: N/P = 0.973 × Mg^2^ - 12.132 × Mg + 45.165). Beyond this point, the N/P ratio increases as Mg content continues to rise.

### 3.4 Plant leaf chemometric PCA analysis

**Figure 10:**
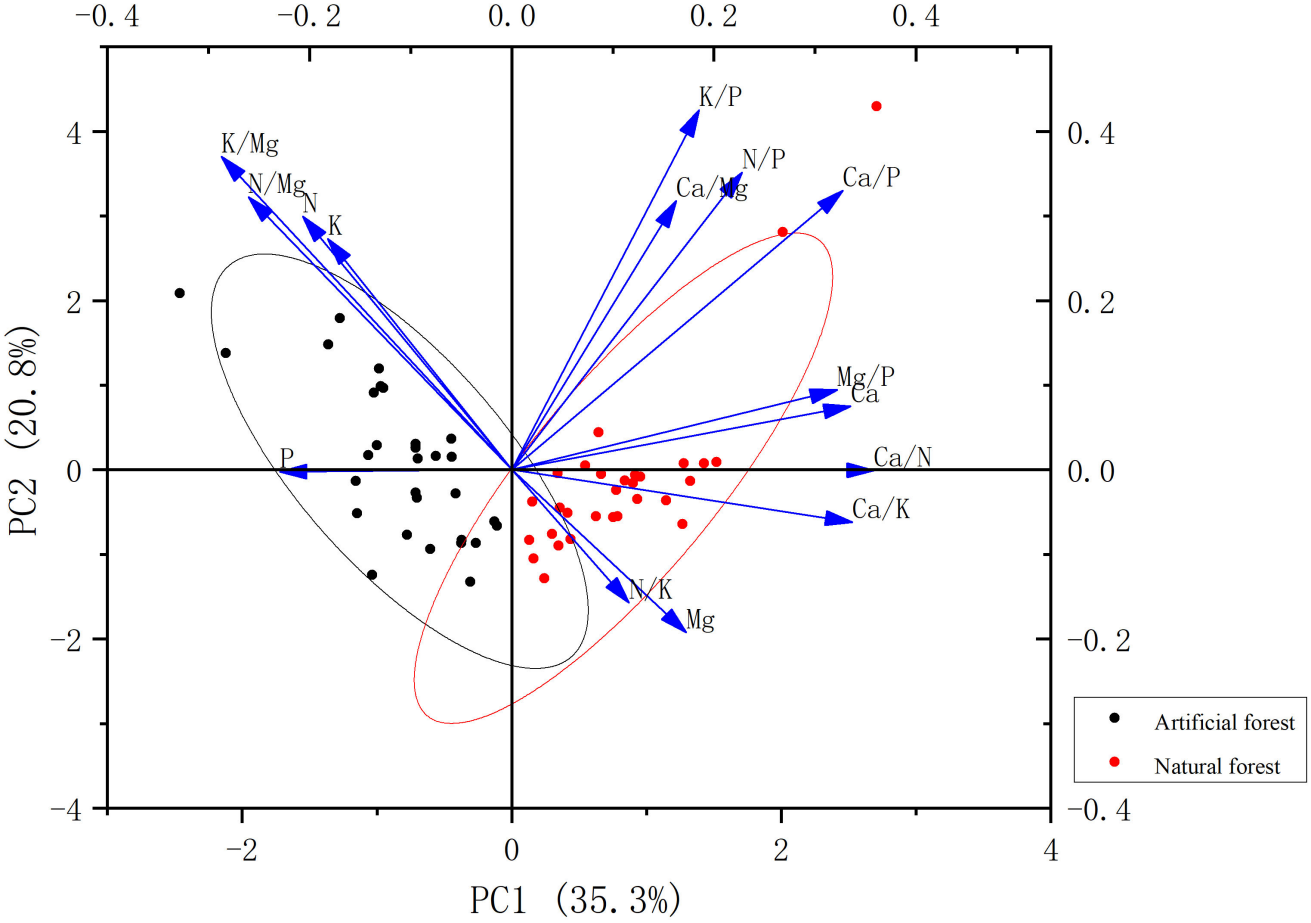
PCA of plant leaf stoichiometry in the artificial forest and natural forest.

Principal component analysis (PCA) was applied to evaluate correlations among various forest types and their associated leaf chemical traits. The PCA results are shown in **Figure 10**. The first two principal components (PC1 and PC2) together explained 56.1% of the total variance, with PC1 accounting for 35.3% and PC2 for 20.8%. The contents of N, P, K, N/Mg, and K/Mg are negatively correlated with PC1, while others are positively correlated with PC1. Variables that contributed most significantly to PC1 included Ca/N, Ca/K, Ca/P, Ca, and Mg/P. The Mg, N/K, and Ca/K contents show a negative correlation with PC2, while others are positively correlated with PC2. K/P, K/Mg, N/Mg, N/P, and N contribute significantly to PC2.

The PCA plot clearly distinguishes plant species according to forest type. Plant samples from artificial forests are positioned closer to the vectors of N- and P-related variables, indicating higher nutrient levels and suggesting the adoption of a fast-growth strategy. In contrast, natural forest plant samples are more strongly associated with Ca- and Mg-related variables, implying a nutrient-conservative or slow-growth strategy.

## 4 Discussion

### 4.1 Basic characteristics of chemical elements in plant leaves

N and P are fundamental components of numerous key biomolecules in plants and play vital roles in maintaining normal growth and physiological function (Lu et al., 2011). In the present study, the average foliar N contents in artificial and natural forests of the coastal sands of northern Hainan Island were 10.9% and 19.1% lower than the mean N content of leaves of terrestrial plants in China (20.2 g/kg) (Han et al., 2005), and 10.4% and 18.7% lower than the global average (20.1 g/kg) (Reich and Oleksyn, 2004). The average foliar P contents in artificial and natural forests were 24.0% and 39.0% lower than the average for Chinese terrestrial plants (1.46 g/kg) (Han et al., 2005), and 37.3% and 49.7% lower than the global average (1.77 g/kg) (Reich and Oleksyn, 2004). Numerous studies have demonstrated that foliar N and P stoichiometry varies significantly among individuals, species, life forms, habitats, and across latitudinal gradients; in some cases, N content may vary up to 30-fold and P content more than 100-fold among individuals (Tian et al., 2018). This large variation may explain the lower N and P concentrations observed in this study relative to national and global reference values. Reich (Reich and Oleksyn, 2004) further suggested that foliar N and P contents tend to decrease with declining latitude. In line with this pattern, the lower leaf N and P levels in the coastal sandy vegetation of northern Hainan Island—especially the more pronounced decrease in P—suggest that tropical regions characterized by high temperature and rainfall are more likely to face phosphorus limitation than N limitation.

K is one of the three essential chemical elements for plants, plays an important role in various physiological and biochemical processes in plants, and is the most abundant cation in plants (Bing-song et al., 2002; Soumare et al., 2022). In the present study, the average foliar K concentrations in artificial and natural forests on the coastal sandy land of northern Hainan Island were notably lower than the national average K content (15.09 g/kg) reported across 660 plant species in China (Qin et al., 2010). This discrepancy may be attributed to the generally low K levels in soils of subtropical regions, where high temperatures and intense rainfall promote leaching and weathering, thereby accelerating K loss from the soil (Liu et al., 2020).

Ca plays a vital role in maintaining the structural integrity of plant cell walls and membranes, and contributes significantly to enhancing leaf resistance to frost damage (McLaughlin and Wimmer, 1999). The average Ca content of plant leaves in the artificial forest and natural forest in coastal sandy land in northern Hainan Island was compared with the average Ca content (15.38 g/kg) reported for 660 plant species across China (Qin et al., 2010), the artificial forest value is substantially lower, while the natural forest value is approximately equivalent. In recent years, increasing soil acidification caused by acid deposition in South China has led to a decline in available soil calcium, posing a growing risk of Ca deficiency in plant leaves (Bo et al., 2000; Jiang et al., 2021). Therefore, long-term monitoring of foliar Ca levels in evergreen broad-leaved forests is essential for the prevention of nutrient deficiency and the maintenance of forest health.

Mg is a central component of chlorophyll and participates in the activation of several enzymes involved in photosynthesis (Guo et al., 2016; Hu et al., 2024). In the present study, the average Mg content in the leaves of artificial and natural forest plants on the coastal sandy land of northern Hainan Island exceeded the typical Mg concentration range (0.5–1.3 g/kg) reported for terrestrial plants (Smidt, 1988). Compared to the average Mg content in leaves of tropical evergreen broad-leaved forest plants in Hainan (4.81 g/kg) (Dongsheng and Lin, 2003), the values observed in this study show no significant difference. Maintaining appropriate levels of Ca and Mg in plant tissues is crucial for ensuring healthy growth and metabolic balance. In this study, Mg exhibited the highest coefficient of variation among the ten examined species, likely due to species-specific differences in Mg uptake and transport. Compared to macronutrients, trace elements such as Mg tend to show greater interspecific variability in plant tissues (White et al., 2018).

For N, P, and K, the absence of statistically significant differences in variance between forest types suggests that these essential macronutrients are more uniformly constrained by the fundamental limitations of coastal sandy soils. The marginal non-significance for calcium (p = 0.059) suggests a potential trend toward greater variability in artificial forests. Compared to natural forests, this model may reflect more diverse management interventions or species selection in artificial forests. The higher variability of magnesium in natural forests (F = 11.323, p = 0.010) strongly supports the hypothesis of greater ecological heterogeneity in these complex ecosystems. This pattern likely reflects the broader functional diversity and species-specific Mg acquisition strategies in natural forests, where different tree species employ varied approaches to Mg uptake and utilization.

Based on the mean values of foliar elemental concentrations across different forest types, substantial differences in nutrient accumulation patterns were observed. These differences can be largely attributed to genetic traits, including variations in morphological structure and physiological mechanisms. As a result, resource utilization efficiency varies considerably among species and forest types, which is well reflected in the stoichiometric characteristics of plant leaves concentration of chemical elements (Li et al., 2010).

The average N/P ratio of natural forests is higher than the national average (16.3) (Han et al., 2005) and the global average (13.8) (Reich and Oleksyn, 2004), while the average value of artificial forests is higher than the global average. The elevated N/P ratios are primarily attributed to the relatively low P concentrations in plant leaves compared to national and global benchmarks. The elevated N/P ratios observed in this study are consistent with a potential for phosphorus limitation in this ecosystem. This pattern aligns with the general tendency toward phosphorus deficiency in highly weathered soils of tropical and subtropical regions. It is important to note, however, that foliar N/P ratios serve as an indicator of plant nutritional status and nutrient use efficiency, This interpretation, based on foliar stoichiometry, warrants further validation through direct soil nutrient assays and nutrient addition experiments. The high N/P ratios could indeed reflect both low soil P availability and efficient plant P-use strategies. Therefore, our findings point to phosphorus as a potentially key limiting nutrient, a hypothesis that should be tested in future studies integrating soil chemistry and nutrient manipulation experiments. It has been shown that the better the soil moisture conditions, the higher the N utilization rate, and in contrast, the N utilization rate of plants is more susceptible to soil moisture and temperature than the P utilization rate (Xing et al., 2000; Li et al., 2024). In our study system, the high N/P ratios observed in natural forest species could reflect both genuine phosphorus limitation and inherent species characteristics shaped by long-term adaptation to coastal sandy soils. Future research should aim to establish ecosystem- and species-specific critical N/P ratios through integrated approaches combining stoichiometric analysis with nutrient addition experiments and physiological measurements.

In artificial forests on the coastal sandy land of northern Hainan Island, a negative relationship was observed between the N/P ratio in plant leaves and Mg content. The fluctuations in N/P were more strongly influenced by changes in phosphorus concentration (**Figure 6**). When foliar Mg content was below 6.24 g/kg, P was the primary driver of N/P variation in this range. At the same time, Mg exhibited a stronger correlation with P than with N, resulting in a decline in the N/P ratio as Mg concentration increased. However, when Mg concentration exceeded 6.18 g/kg, the N/P ratio began to increase with rising Mg levels (**Figure 8**). These findings suggest that fluctuations in the N/P ratio are not solely dependent on N and P contents but may also be modulated by variations in Mg concentration. Differences in Mg cycling may influence P uptake and utilization to some extent; however, this relationship is likely affected by a range of factors including soil conditions, plant species, and broader environmental variables.

In both artificial and natural forests of the coastal sandy area in northern Hainan Island, the K/P ratio in plant leaves indicated that the growth and development of the ten studied species were not limited by K. This finding is consistent with the results of (Yan et al., 2011; Zhang et al., 2018), who reported that plant growth in harsh environments is generally less constrained by K availability. The reduced susceptibility to K limitation may be attributed to adaptive survival strategies that have evolved over long periods in response to environmental stress. K plays a critical role in enhancing plant drought tolerance and disease resistance. In nutrient-poor soils typical of tropical coastal regions, plants may selectively absorb and accumulate higher levels of K to strengthen their resistance to environmental stressors.

### 4.2 Relationship between chemical elements in plant leaves

The correlation between N, P, and K in the leaves of plants from artificial and natural forests on the coastal sandy areas of northern Hainan Island suggested that these plants may have evolved mechanisms to optimize the synergistic use of N, P, and K (Zhang et al., 2012). As alkaline-earth metals, Ca and Mg also exhibit comparable chemical behaviors and biogeochemical properties. Some international studies have suggested a correlation between Mg and N, possibly because higher nitrogen availability enhances photosynthesis, thereby increasing chlorophyll synthesis where Mg is a key component (De Vries et al., 2003; Neugebauer et al., 2018). However, other studies have found no significant relationship between Mg and N (Osaki et al., 2003; Xu et al., 2024), which aligns with the findings of this study.

The observed stronger correlations among chemical element concentrations in plant leaves from artificial forests, compared to natural forests, may be explained by several mechanisms. First, the management practices in artificial forests, including potential fertilization and irrigation, likely create more homogeneous soil nutrient conditions (Hao et al., 2019). This reduced environmental variability may lead to more synchronized nutrient uptake patterns among species, thereby strengthening elemental correlations in leaf tissues. Second, the simplified species composition in artificial forests, often dominated by few selected species with similar nutrient requirements, could result in more consistent nutrient allocation strategies across the community. In contrast, the high species diversity in natural forests encompasses a wider spectrum of nutrient use strategies and physiological adaptations, potentially diluting overall correlation strength through niche differentiation and resource partitioning (Wang et al., 2019). Third, the selection of fast-growing species for artificial forests might favor plants with coordinated nutrient uptake mechanisms optimized for rapid growth, whereas natural forests include species with diverse life history strategies and nutrient conservation mechanisms. Although these mechanisms provide reasonable explanations for our observations, future research needs to directly measure soil nutrient heterogeneity, species specificity, and forest type management practices to test these hypotheses and elucidate potential driving factors for observed correlation patterns.

The normal functioning of plant physiological processes relies on the absorption and utilization of mineral elements in certain ratios and the maintenance of nutrient balance. This balance may be disrupted by limitations in elemental availability or by fluctuations in habitat conditions (Ordoñez et al., 2009). Correlations among elemental concentrations in plant tissues can vary significantly across different regions and ecosystems. Therefore, the interpretation of elemental relationships in leaves should not rely solely on quantitative statistical analysis, but must also incorporate mechanistic insights into plant nutrition, soil nutrient dynamics, and species-specific physiological tolerance. In this study, the leaf N, P, K, Ca, and Mg varied among tree species without showing a consistent trend. This variability may reflect differences in elemental requirements and utilization strategies among species during growth and development (Zhang et al., 2010; Wu et al., 2020). Such interspecific differences are influenced not only by genetic traits but also by environmental factors such as soil nutrient availability, water conditions, and light regimes (Meng et al., 2019; Jiashalaiti et al., 2024). At the same time, the chemical composition of vegetation and its dynamics often follow broader ecological laws, exhibiting certain patterns of universality. The present study reflects both the regional characteristics of foliar elemental composition in tropical coastal sandy vegetation of Hainan and the general patterns of elemental distribution and interaction among plant species concentration of chemical elements. Overall, plant nutrient demand is a complex process jointly influenced by meteorological, edaphic, and biological factors. Given the high biodiversity, strong biogeochemical cycling, and complex ecological processes in tropical ecosystems, future research and management should adopt species-specific and context-dependent approaches. Tailored restoration technologies for tropical coastal sandy vegetation should be developed based on plant functional types, site conditions, and specific ecological objectives.

### 4.3 Plant nutrient utilization strategies

The principal component analysis (PCA) successfully captured 56.1% of the total variance in the foliar stoichiometric dataset with the first two axes. While this level of explained variance is common in ecological studies dealing with complex, multi-species systems and is sufficient to identify the primary stoichiometric gradients separating forest types, it also indicates that additional factors contribute to leaf elemental variation. The remaining variance (43.9%) is likely attributable to a combination of unmeasured factors intrinsic and extrinsic to the plants. These may include: (1)Species-specific physiological traits: Genetic differences in nutrient uptake kinetics, allocation patterns, and internal recycling efficiencies among the ten dominant species, which are not fully captured by forest type alone.(2)Microsite heterogeneity: Fine-scale variations in soil moisture, microbial activity, and root competition within plots, which can create localized nutrient hotspots or limitations.(3)Unmeasured plant functional traits: Traits such as specific leaf area (SLA), leaf dry matter content (LDMC), or photosynthetic rates, which are closely linked to the leaf economics spectrum and could provide a more mechanistic explanation for nutrient strategies. Nevertheless, the clear separation of artificial and natural forest samples along the primary PCA axis (PC1), which is strongly driven by the trade-off between N/P and Ca/Mg, provides robust support for the existence of contrasting nutrient utilization syndromes at the community level. Our interpretation of growth strategies focuses on this dominant and ecologically meaningful pattern. Future studies that integrate a broader suite of plant functional traits, soil microbial data, and finer-scale environmental measurements would be valuable to account for a greater proportion of the observed variance and refine our understanding of the mechanisms underlying these strategies.

Differences in nutrient utilization strategies among plant species from different forest types are primarily reflected in their ability to acquire and efficiently utilize key nutrients such as N and P. Fast-growing species typically allocate more biomass to stems and leaves to enhance light acquisition, and exhibit higher photosynthetic and respiration rates supported by elevated N levels. In contrast, slow-growing species generally allocate more biomass to root systems to enhance nutrient uptake and adapt to nutrient-poor soils. Slow-growing species are more oriented toward long-term survival and stable resource use, often enhancing nutrient uptake efficiency and minimizing nutrient loss to persist in low-nutrient environments (Reich et al., 1997; Wright et al., 2004; Read et al., 2014; Wan et al., 2023). These differences indicate that nutrient utilization and partitioning are regulated jointly by external nutrient limitations and intrinsic physiological traits. The PCA results show that artificial forest samples were associated with higher nitrogen and phosphorus scores, while natural forest samples were associated with higher calcium and magnesium scores. This pattern suggests that dominant species in artificial forests may align with rapid growth strategies, while species in natural forests may align with slow growth strategies. It is worth noting that this explanation is inferred based on foliar stoichiometry and its good correlation with plant functional traits. Although this method has been widely applied in comparative ecology, and our findings are supported by previous studies in similar systems (Qi et al., 1996; Huang, 2004; Wu et al., 2023), future research can combine growth rate, photosynthetic capacity, and resource utilization efficiency to clearly validate these nutrient related strategy classifications. It has also been reported that fast-growing species are less tolerant to drought stress than slow-growing species (Piper, 2011; Mitchell et al., 2013; Zhang et al., 2020; Li et al., 2022). Given that coastal sandy soils are loose with poor water-holding capacity, they are prone to water stress. Therefore, by analyzing the PCA positions of plant species from different forest types and their relationships with stoichiometric vectors, it is possible to preliminarily infer their nutrient utilization strategies and growth types. This has important implications for designing forest types better suited to coastal sandy environments. Some artificial forest samples were classified as fast-growing species, and some natural forest samples as slow-growing species, suggesting that these patterns may also be influenced by species-specific traits. Further studies integrating ecological background knowledge, long-term field observations, and in-depth trait-based analysis are necessary to validate these findings and optimize forest management strategies.

The foliar nutrition data provided in this study were obtained from sampling events during the peak growth period (July). This method is common in comparative plant stoichiometry studies because it captures vegetation in active growth and relatively stable nutrient states, but it does not take into account the potential seasonal dynamics of nutrient concentrations. Seasonal fluctuations in temperature, precipitation, and plant phenology can affect nutrient absorption, transport, and reabsorption. Therefore, the research results mainly provide a comparison of nutritional strategies between forest types under peak physiological activity. Future research combining multi seasonal sampling will help clarify the temporal stability of these patterns and understand how nutrient cycling and reabsorption efficiency affect changes in these coastal sandy forests.

## 5 Conclusions

This study reveals distinct differences in leaf nutrient characteristics between artificial and natural forests in the coastal sandy areas of northern Hainan Island. (1) Leaf N, P, and K concentrations were significantly higher in artificial forests, while Ca and Mg concentrations were higher in natural forests. These differences may be attributed to variations in soil nutrient availability and plant physiological traits. (2) Vegetation in tropical coastal sandy areas is primarily limited by phosphorus (P), with K/P *>* 3.4 indicating an absence of potassium limitation. Calcium, nitrogen, and magnesium are relatively sufficient, while P limitation may be associated with Mg dynamics and is regulated by multiple interacting factors. (3) Stronger inter-element correlations were observed in artificial forests, likely due to monoculture, standardized management, and stable nutrient inputs. The fluctuation of the N/P ratio was influenced by Mg levels, suggesting that Mg cycling may impact P utilization. (4) Principal component analysis revealed that artificial forest samples scored higher on N- and P-related indicators, suggesting a fast-growth strategy, while natural forests showed higher scores on Ca and Mg, indicating a slow-growth strategy.

For artificial forests, precise fertilization should be applied to regulate nutrients while avoiding excessive fertilization that can lead to soil salinization. Currently, artificial forests are mostly composed of monocultures species. It is recommended to introduce companion tree species to maintain the sand fixation and rapid growth functions of artificial forests, while improving soil and enhancing ecological stability through companion tree species.

For natural forests, human interference should be strictly controlled, soil nutrient reservoirs should be protected, and behaviors such as excessive logging and understory cultivation should be prohibited. When replanting natural forests, priority should be given to selecting tree species that are suitable for the Ca/Mg enrichment environment in sandy areas, and avoiding the introduction of foreign tree species with high P demand.

Overall, this study provides new insights into the nutrient status of artificial and natural forest vegetation in tropical coastal sandy areas. These findings are valuable for evaluating vegetation restoration effectiveness, improving ecological health assessments, and guiding sustainable management practices in tropical coastal sandy ecosystems.

## Data Availability

The original contributions presented in the study are included in the article/supplementary material. Further inquiries can be directed to the corresponding author.
